# Risk of avoidant/restrictive food intake disorder in patients with inflammatory bowel disease: predictive value of disease phenotype, disease activity and food literacy

**DOI:** 10.1186/s40337-023-00936-3

**Published:** 2023-11-28

**Authors:** Tingting Yin, Wenjing Tu, Yiting Li, Min Yang, Lina Huang, Sumin Zhang, Guihua Xu

**Affiliations:** 1 Nursing School, Nanjing University of Chinese Medicine, Nanjing, 210023 China; 2 Anorectal Department, Nanjing City Hospital of Traditional Chinese Medicine, Nanjing, 210006 China

**Keywords:** Inflammatory bowel disease, Avoidant/restrictive food intake disorder, Food literacy

## Abstract

**Background:**

Avoidant/Restrictive Food Intake Disorder (ARFID) is a newly described eating disorder. Adequate levels of food literacy allow individuals to have adequate food choices. This study aimed to assess the prevalence of ARFID and the level of food literacy in patients with inflammatory bowel disease (IBD) and to analyse the correlation between ARFID and food literacy.

**Method:**

This cross-sectional study screened for ARFID and assessed food literacy levels in patients with IBD attending four tertiary hospitals in China. ARFID risk was measured using the Nine Item Avoidant/Restrictive Food Intake Disorder Screen (NIAS). Food literacy was assessed using the Food Literacy Evaluation Questionnaire (Chinese version, FLEQ-Ch).The relationship between individual NIAS scores and food literacy variables was analysed to assess which food literacy aspect is positively or negatively associated with NIAS scores. Stepwise linear regression analysis was performed to identify the possible predictors of NIAS scores in patients with IBD.

**Result:**

A total of 372 IBD subjects completed the NIAS and FLEQ-Ch. The overall mean NIAS scores for the IBD cohort was 28.16 ± 8.03 (*p* < 0.01), and of the 372 participants, 123 (32.5%) had positive ARFID risk scores (≥ 10 NIAS-picky eating, ≥ 9 NIAS-poor appetite, and ≥ 10 NIAS-fear of negative consequences).The NIAS scores were inversely associated with food literacy levels (β =  − 0.299; *p* < 0.01).Disease phenotype, disease activity, and food literacy in patients with IBD provided valuable predictive insights for avoiding positive outcomes in ARFID.

**Conclusion:**

This study shows that the risk of ARFID in the cohort of patients with IBD is associated with their inadequate food literacy levels. Therefore, this study supports the notion that patients with IBD should be assessed for food literacy regardless of whether they are currently diagnosed with ARFID. Specifically, for early identification of those at risk for ARFID in IBD, disease phenotype, disease activity, and food literacy should be routinely considered in clinical practice.The food literacy awareness of patients must be investigated and improved to predict the risk occurrence of ARFID and encourage healthy eating behaviour.

## Abstract (plain language summary)

Excessive dietary restriction is common in patients with inflammatory bowel disease (IBD) and in severe cases can lead to Avoidant/Restrictive Food Intake Disorder (ARFID).ARFID is a new eating disorder that has been proposed in recent years. Studies have shown that food literacy can influence patients’ eating behaviour to some extent. Research on the risk factors associated with ARFID is necessary to better understand why people with IBD develop ARFID and to inform programmes aimed at reducing these risk factors. In our study, we found that disease phenotype, disease activity, and food literacy in patients with IBD provided valuable predictive insights for avoiding positive outcomes in ARFID. These factors can provide valuable perspectives for healthcare providers and patients at this critical juncture in the disease dietary management process. Therefore, this study supports the notion that patients with IBD should be assessed for food literacy regardless of whether they are currently diagnosed with ARFID. Specifically, for early identification of those at risk for ARFID in IBD, disease phenotype, disease activity, and food literacy should be routinely considered in clinical practice.

## Introduction

Ulcerative colitis (UC) and Crohn's disease (CD) are the dominant clinical phenotypes of inflammatory bowel disease (IBD) and characterised by chronic recurrent intestinal inflammation. The global prevalence of IBD has increased significantly in recent years, especially in industrialised countries such as China [1]. One reason for this phenomenon could be dramatic changes in diet and lifestyle. The Western-style diet can negatively affect homeostasis in the gut microbiome and induce episodes of UC and CD [2, 3]. To manage the symptoms associated with the disease, many patients with IBD alter their diet often by avoiding or restricting specific foods or food groups. The Asian Working Group guidelines recently confirmed that a healthy diet rich in fruits, vegetables and n-3 fatty acids and low in n-6 fatty acids, plays a preventive role in the onset of IBD [4]. This complicated connection has led patients with IBD to seek dietary solutions for disease management. However, current guidelines do not include evidence-based dietary recommendations [5, 6]. Previous research found that half of the patients with IBD did not receive any dietary advice and therefore choose to follow general advice, which is not always based on scientific advice [7, 8]. Owing to the lack of easily accessible and conclusive dietary advice, patients with IBD taking an independent, unsupervised approach to disease control through diet may develop restrictive eating behaviours that lead to inadequate nutritional intake and increased risk of malnutrition [9–11]. Previous studies have found that up to 28%–89% of patients with IBD have some degree of dietary contraindications, and 41–93% have restrictive eating behaviours [12, 13].

Avoidant/Restrictive Food Intake Disorder (ARFID) is an eating or feeding disorder that incorporates three different eating patterns of symptoms: (a) avoidance of food due to its sensory properties (e.g. picky eating), (b) poor appetite or limited interest in diet and (c) fear of the negative consequences of eating (e.g. choking or vomiting) [14]. ARFID can be diagnosed in individuals of any age or developmental level and is characterised by restrictive eating that leads to weight loss, nutritional deficiencies, supplement dependence and/or severe psychosocial disorders and cannot be attributed solely to body type and weight issues or medical comorbidities. The prevalence of ARFID may be high in patients with IBD [15], and its onset may be due to concerns about the negative consequences of gastrointestinal symptoms caused by eating [16]. Werlang et al. [17] found that the overall prevalence of ARFID was 10.2% in patients with IBD (n = 98), and the prevalence of ARFID was higher in CD than in UC. Harer et al. [18] also found an increased prevalence of IBD in patients with ARFID, with 4 out of 28 patients (14%) being diagnosed with IBD. Patients with IBD are often influenced by past or expected gastrointestinal experiences, and those who exhibit active symptoms and/or inflammation are likely to screen positive for ARFID risk; therefore, these patients have difficulty integrating healthy eating into their daily lives and may perpetuate ARFID due to poor food literacy [19]. The occurrence of ARFID is a major challenge in ensuring adequate dietary nutrition in IBD diet management. Misinformation about the effects of food and nutrition may be detrimental for patients with IBD because of its significant effect on the potential risk of social life and nutritional deficiencies [20]. On the basis of the above premise, adequate food choices appear to be a concrete tool for patients to control disease progression.

Food literacy is a developing concept that first appeared in public health literature in 2001 and has been used in health and education research since 2010. Early studies defined food literacy as ‘an individual's ability to access, process and understand basic information about food and nutrition and to use that information to make appropriate health decisions’ [21, 22]. The most widely cited definition of food literacy is that proposed by Vidgen and Gallegos, who described it as ‘the collection of interrelated knowledge, skills and behaviours needed to plan, manage, select, prepare and eat food to meet needs and determine food intake’ [23]. Food literacy emphasises the importance of these applied skills, such as selecting, preparing and eating food; applying food-related information; and participating in complex food systems [24]. A previous study confirmed a widespread lack of food literacy among the IBD population in Italy [19]. Patients with insufficient food literacy struggle to make correct food choices, which has a negative impact on their health status and quality of life [19]. Limited levels of food literacy alter patients’ ability to assess and meet their individual nutritional needs, and the great risk of misconceptions about healthy food choices leads to unhealthy eating behaviours [25]. To our knowledge, the exact relationship between food literacy and ARFID in Chinese IBD subjects has not been investigated.

This study aimed to investigate the level of food literacy in patients with IBD and explore its relationship with ARFID. Our research is the first to reveal the impact of food literacy degree on the ARFID of patients with IBD. Additionally, we analysed the relationship between individual the Nine Item ARFID Screen (NIAS) scores and disease and sociodemographic variables to assess which food literacy aspects are positively or negatively associated with ARFID levels.

This study aimed to identify the main factors associated with ARFID to guide future healthy eating behaviours and nutrition information strategies, thereby improving nutrition awareness and health outcomes in patients with IBD.

## Materials and methods

### Study design and participant recruitment

This cross-sectional study was conducted at IBD centres at four tertiary hospitals in Nanjing, China. Inclusion criteria were as follows: aged 18 years or above and diagnosed with IBD (confirmed by a combination of endoscopic, radiological, biochemical and histological investigations). Eligible patients were invited to participate in this ongoing study since October 2022. Exclusion criteria were as follows: (1) pregnant or lactating women; (2) with cognitive dysfunction and/or severe mental illness; (3) with celiac disease, known food allergies or surgery that affect gastroenteric function; and (4) followed any special diet or dietary pattern (IBD-specific diets, such as the CDED diet and low FODMAP diet, vegetarian, vegan or related to particular religious or social traditions). Prior to study inclusion, written informed consent was obtained from all eligible IBD subjects. This study followed the principles of the Declaration of Helsinki, was approved by the Ethics Committee of the affiliated hospital and the Institutional Review Committee (project approval number KY2022029), and completed registration in the Chinese Clinical Trials Registry (ChiCTR2200064943, accessed on 24 October 2022).

### Sample size

Owing to the lack of data on the prevalence of ARFID among Chinese patients with IBD, a pilot trial was conducted to calculate the prevalence using the Chinese version of the NIAS (a positive score on any NIAS subscale (≥ 10 NIAS-picky eating, ≥ 9 NIAS-poor appetite and ≥ 10 NIAS-fear of negative consequences) scale and food literacy in patients with IBD before the start of the study. The results showed that 20% (23/116) of the patients tested positive for ARFID. In previous studies, the prevalence of ARFID is 12–21% among patients with various gastrointestinal disorders [15, 18, 26]. Pre-experimental prevalence was included in the known range. Therefore, the prevalence of 21% was used to calculate the sample size using a single proportional formula (n = Z_α/2_^2^ pq/d^2^) q = (1 − p), Z_α/2_ = confidence interval (CI) of 95% = 1.96, d = margin of error of 5% = 0.05.The estimated sample size was 255 IBD subjects.

### Data collection and measurements

The first part was data collection through the questionnaire. All data collection and processing are anonymous. The subjects were investigated for their socio-demographic characteristics, such as gender, age, weight, height, education level, occupation, disease subtypes (CD or UC) and disease activity. Disease activity was assessed using the Harvey–Bradshaw Index (HBI) for CD and the Mayo scale for UC after accurate clinical assessment by an experienced IBD clinician. Active IBD-related symptoms were defined as HBI score > 4 for patients with CD or Mayo scale score ≥ 2 for patients with UC.

### The nine item avoidant/restrictive food intake disorder screen (NIAS)

NIAS is a 9-item self-reported questionnaire that assesses avoidant/restrictive eating patterns and comprises three subscales: the picky eating subscale that measures sensory aversion to food (e.g. ‘I dislike most foods that other people eat’), the poor appetite subscale that measures lack of interest in eating or food (e.g. ‘Even when I am eating foods I really like, it is hard for me to eat a large enough volume at meals’) and the fear of negative consequences subscale that measures fear of aversive consequences as a consequence of eating (e.g.’I avoid or put off eating because I am afraid of GI discomfort, choking, or vomiting’). The questions are based on a 6-point Likert scale, and individuals respond to each question on a scale from 0 (Strongly Disagree) to 5 (Strongly Agree). Subscales are each scored on a scale from 0 to 15, with higher scores indicating higher levels of each metric (picky eating, poor appetite, and fear of negative consequences). All items may also be summed to calculate a total score, ranging from 0 to 45, with higher scores indicating higher levels of avoidant/restrictive eating broadly [27]. In this study, we used the Chinese Version of the Nine Item ARFID Screen to investigate the dietary behaviours of patients with IBD [28].Cronbach alphas in the current sample were 0.77, 0.79, and 0.81 for the picky eating, poor appetite, and fear of negative consequences subscales, respectively. Burton et al. [29] recommend identifying possible cases of ARFID by a positive screen on any NIAS subscale (≥ 10 NIAS-picky eating, ≥ 9 NIAS-poor appetite, and ≥ 10 NIAS-fear of negative consequences). In this study, we considered participants who met the any NIAS subscale (≥ 10 NIAS-picky eating, ≥ 9 NIAS-poor appetite, and ≥ 10 NIAS-fear of negative consequences) as having a ARFID risk.

### Assessment of food literacy

Given that the definition and scope of food literacy concepts vary depending on the context of the study, a variety of methods that can be used to measure them. One such approach is the Food Literacy Evaluation Questionnaire (Chinese version, FLEQ-Ch) by Qian et al. [30]., which measures practical food skills and knowledge in a clear and concise manner and applies to the Chinese population. Food literacy levels were assessed using the FLEQ-Ch, which consists of 15 items with response options on a 4-point Likert scale (1 = ‘not at all/never’ to 4 = ‘yes/always’). The first factor named ‘(Foods) Planning and management’ reflects ‘foods planning and management’ and includes items 1–7 (e.g.’Make a list before you go shopping’); the second factor named ‘(Foods) Selection’ reflects ‘when selecting foods, whether consider price, nutritional content, etc.’ and includes items 8–10 (e.g.’Use nutrition information panel to make food choices’); and the third factor named as ‘(Foods-making) Attitude’ reflects the positive attitude of making foods and includes items 11–15 (e.g.’Change recipes to make them healthier’). High FLEQ-Ch scores indicate levels of food literacy, and the mean FLEQ-Ch score is the mean of the individual scores of 15 items. All items may be summed to calculate a total score ranging from 15 to 60, and the three dimension scores are (Foods) Planning and management 7–28, (Foods) Selection 3–12 and (Foods-making) Attitude 5–20. In this study, Cronbach's alpha = 0.915, indicating that the assessment of food literacy had high internal consistency.

### Statistical analysis

Statistical analyses were conducted using SPSS 26.0 and R statistical (corrplot package) programs. Chi-square test or Fisher exact probability method was used to compare counting data. Shapiro–Wilk test was used to determine whether the continuous variables fit the normal distribution. Parametric data were summarised as mean ± standard deviation (SD) or percentage. Univariate analysis included NIAS scores as the dependent variable and sociodemographic as independent variables. Bivariate analysis using Pearson's correlation test was used to assess the relationship between NIAS scores and continuous variables. Meanwhile, student t-test and ANOVA F tests were adopted to assess the association between categorical variables with two or more levels and the NIAS scales. Kruskal–Wallis test was applied to examine significant differences in NIAS dimensions and NIAS scores across clinical characteristics (such as gender, IBD type, body mass index [BMI] and disease activity) and food literacy. A stepwise method was applied to simultaneously remove the weakest correlated variables and come up with a model that best explains the distribution. Stepwise regression analysis models were used to assess the association between sociodemographic and food literacy and NIAS score. All variables with *p* < 0.05 in the bivariate analysis were included in the model to eliminate potential confounding factors. The criterion for entry was 0.05, and 0.10 was used to exclude variables. A two-sided *p*-value of < 0.05 was considered statistically significant. Cronbach’s alpha was used to assess the reliability of the scales.

## Results

### Study population

**Fig. 1:**
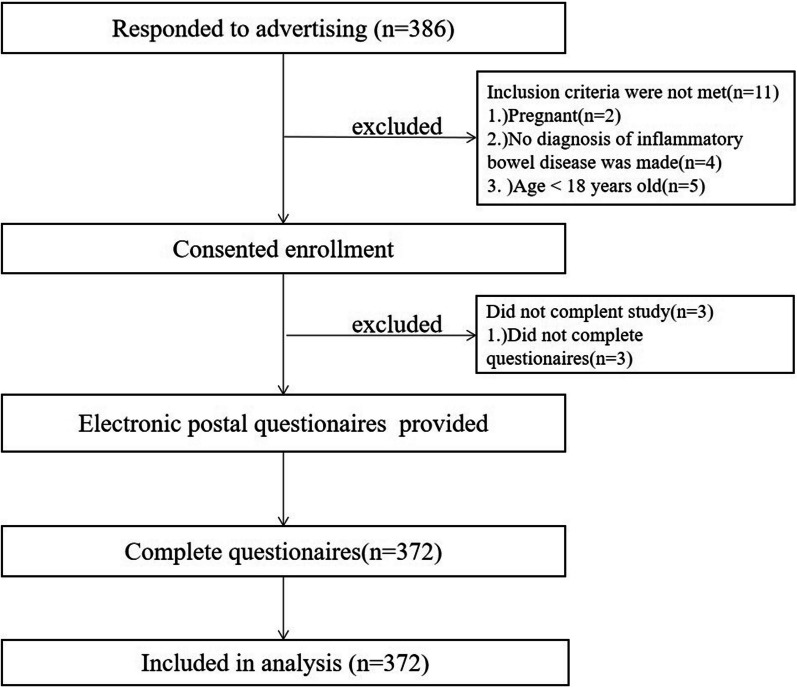
Flow chart of the study

A total of 386 participants were enrolled in this study. Among them, 11 participants did not meet inclusion criteria and 3 had partial or uncompleted questionnaires. With a response rate of 96%, 372 participants who completed both parts of the questionnaire were included in the analysis (Fig. 1). Among them, 245 were male (65.9%) and 127 were female (34.1%). The average age was 38.82 years. More than half of the participants (60.0%) had UC, and 160 (40.0%) had CD. Most of the respondents (90.6%) lived in urban areas, the remaining (9.4%) lived in suburban or rural areas. The detailed demographic information and statistical description of the participants are presented in Tables 1 and 2.

**Table 1:** Statistical description of categorical variables and the NIAS scores of study samples (n = 372)

Categorical variables	N (%)	The NIAS scores				
		M ± SD	95% CI for mean		Statistic parameter	*p-*value
			Lower	Upper		
Total	372 (100)				–	–
Gender					0.575^a^	0.753
Male	245 (65.9)	28.07** ± **8.11	27.05	29.09		
Female	127 (34.1)	28.35** ± **7.91	26.96	29.74		
*Disease phenotype*					1.894^a^	< 0.01
Ulcerative colitis	212(60.0)	25.42** ± **7.77	24.36	26.47		
Crohn’s disease	160(40.0)	31.81** ± **6.85	30.74	32.88		
*Educational level*					0.101^b^	0.959
Master's or above	8(2.2)	29.00** ± **5.29	24.58	33.42		
Junior college or bachelor	326(87.6)	28.19** ± **8.00	27.32	29.06		
Secondary Education	33(8.9)	27.94** ± **8.12	25.06	30.82		
Primary school or below	5(1.3)	26.60** ± **13.90	9.34	43.86		
*Occupation*					2.999^b^	0.051
Employee/student	254(68.3)	28.71** ± **7.98	27.73	29.70		
freelancer	75(20.2)	27.81** ± **8.15	25.94	29.69		
Retirement	43(11.5)	25.53** ± **7.74	23.15	27.92		
*Living place*					1.300^b^	0.274
Urban	337(90.6)	28.35** ± **7.94	27.50	29.20		
Suburban	19(5.1)	27.32** ± **8.17	23.38	31.25		
Rural	16(4.3)	25.19** ± **9.61	20.07	30.31		
*Clinical disease activity (Mayo/HBI)*					0.459^a^	< 0.01
Remission	224(60.2)	25.86** ± **7.64	24.88	26.84		
Active disease	148(39.8)	31.66** ± **7.44	30.41	32.90		

ARFID, avoidant/restrictive food intake disorder; NIAS, Nine Item avoidant/restrictive food intake disorder Screen; HBI, Harvey Bradshaw Index; M, mean; SD, standard deviation; CI, Confidence Interval

^a^Independent t-test

^b^One-way analysis of variance

**Table 2:** Statistical description of metric variables

Metric variables	M ± SD	95% CI for mean	
		Lower	Upper
Age	38.82** ± **14.7	37.36	40.36
BMI	21.09** ± **3.12	20.77	21.41
NIAS(Score range: 9–45)	28.16** ± **8.03	27.35	28.98
Picky eating domain (Score range: 3–15)	8.56** ± **3.32	8.22	8.90
Poor appetite domain (Score range: 3–15)	9.08** ± **3.15	8.76	9.40
Fear of negative consequences domain(Score range:3–15)	10.53** ± **3.34	10.19	10.87
FLEQ-Ch (Score range: 15–60)	31.69** ± **9.84	30.69	32.69
Planning and management domain (Score range: 7–28)	13.85** ± **5.11	13.33	14.37
Selection domain (Score range: 3–12)	6.25** ± **2.42	6.00	6.49
Attitude domain (Score range: 5–20)	11.59** ± **3.57	11.19	12.00

BMI, Body mass index; NIAS, Nine Item avoidant/restrictive food intake disorder Screen; Picky eating: subscale from NIAS; Poor appetite, subscale from NIAS; Fear of negative consequences, subscale from NIAS; FLEQ-Ch, the Chinese-adapted Food Literacy Questionnaire; Planning and management, subscale from FLEQ-Ch; Selection, subscale from FLEQ-Ch; Attitude, subscale from FLEQ-Ch; M, mean; SD, standard deviation; CI, Confidence Interval

### Statistical description of the NIAS scores

The overall mean NIAS score for the IBD cohort was 28.16 ± 8.03, and 123 out of 372 (32.5%) participants had a positive ARFID risk score. Examination of restricted eating pattern domain revealed that the IBD participants reported mean picky eating behaviour scores of 8.56 ± 3.23, poor appetite 9.08 ± 3.15 and fear of negative consequences 10.53 ± 3.34. The mean scores were higher than the reported NIAS reference scores, particularly due to fear of eating. Of the 148 patients with positive ARFID scores on the NIAS, 36 patients with aversive consequence fear manifestations had active IBD (Table 3).

**Table 3:** Comparison of avoidant/restrictive food intake disorder risk score by clinical disease activity

Characteristic	Picky eating domain	Poor appetite domain	Fear of negative consequences domain	Positive ARFID risk screen	Negative ARFID risk screen	*p*-value
Clinical disease activity^c^						< 0.01
Remission (n = 224)	38 (17.0%)	70 (31.3%)	87 (38.9%)	87 (38.84%)	137 (61.2%)	
Active disease (n = 148)	23 (15.5%)	36 (24.3%)	36 (24.32%)	36 (24.32%)	112 (75.7%)	

ARFID, avoidant/restrictive food intake disorder

c: Active inflammatory bowel disease-related symptoms were defined as Harvey-Bradshaw Index score > 4 for patients with Crohn's disease or Mayo scale score ≥ 2 for patients with ulcerative colitis

### Statistical description of the food literacy scores

The overall mean food literacy score for the IBD cohort was 31.69 ± 9.84 (*p* < 0.01). In the examination of the three domains of food literacy, the IBD participants reported mean planned and managed food scores of 13.85 ± 5.11 (*p* < 0.01), choice mean food score of 6.25 ± 2.42 (*p* < 0.01) and foods-making attitude of 11.59 ± 3.57 (*p* < 0.01).

### Statistical description of the factors relevant to the NIAS scores

**Fig. 2:**
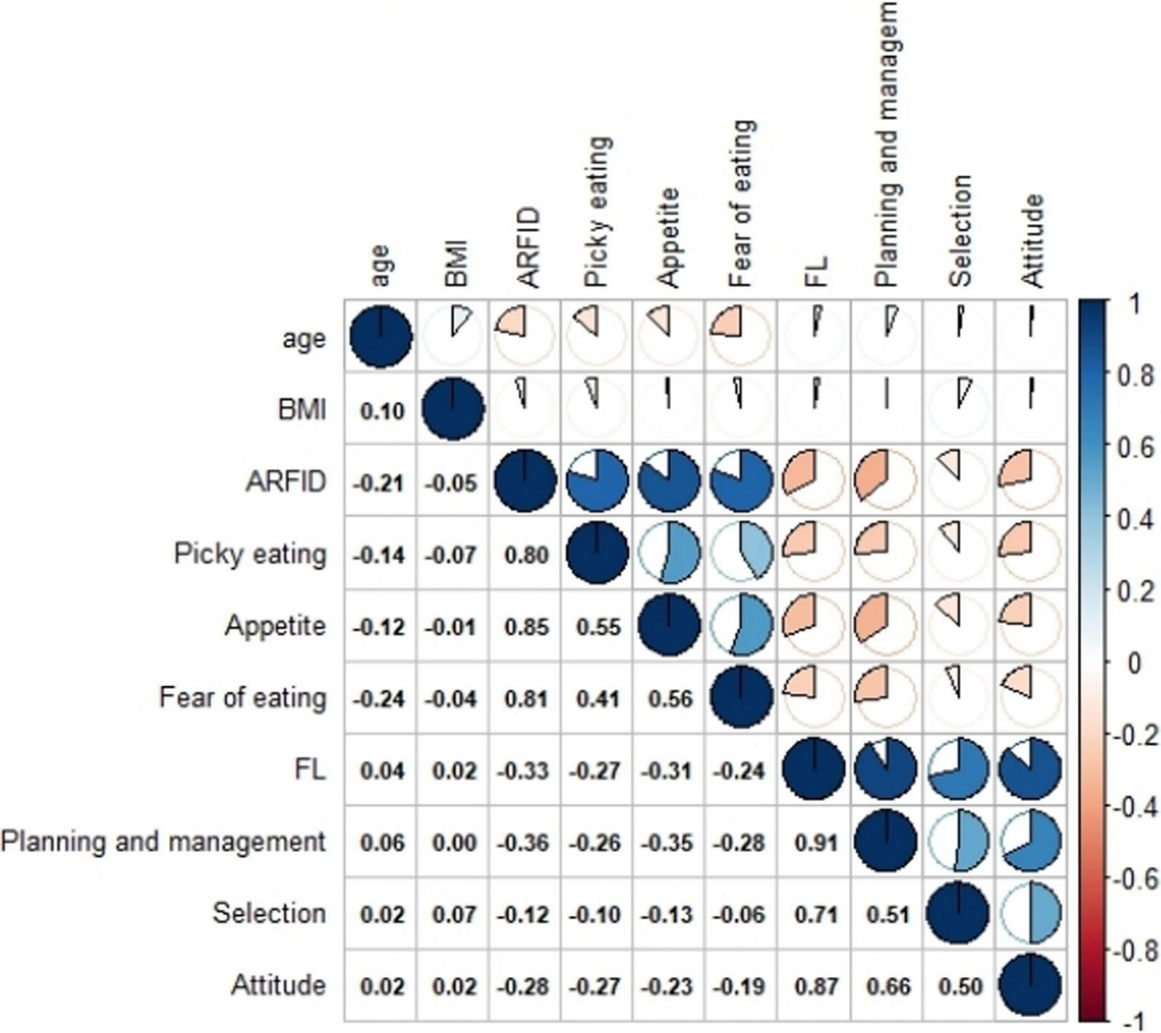
Correlations between the studied variables among the patients with IBD (n = 372). *Note:* BMI: Body mass index; ARFID: Nine Item avoidant/restrictive food intake disorder Screen (NIAS) scores; FL: the Chinese-adapted Food Literacy Questionnaire (FLEQ-Ch) scores; Picky eating: subscale from NIAS; Appetite:subscale from NIAS; Fear of eating: subscale from NIAS; Planning and management: subscale from FLEQ-Ch; Selection: subscale from FLEQ-Ch; Attitude: subscale from FLEQ-Ch

The respondents who were female (28.35 ± 7.91), with CD (31.81 ± 6.85), employee or student(27.81 ± 7.98), had a junior college or bachelor (28.19 ± 8.00), master's degree or above (29.00 ± 5.29), had active disease (31.66 ± 7.44) or lived in the urban area (28.35 ± 7.94) showed higher NIAS scores than the average. Meanwhile, the residents who had UC (25.42 ± 7.77), retired (25.53 ± 7.74), remission (25.86 ± 7.64) or lived in the rural area (25.19 ± 9.61) showed low scores of NIAS. Univariate analysis revealed the limited correlations of NIAS score with gender, educational level, occupational status, residence and education level; in this case, statistically significant difference was not reached (*p* > 0.05) (Table 1). ARFID was associated with food literacy, disease phenotype and disease activity. CD and UC differed significantly within each domain, with higher scores found in all CD domains compared with those in UC domains. Food literacy scores were negatively correlated with NIAS scores; the patients with IBD with reduced food literacy scores had a significantly increased likelihood of positive risk screening for ARFID (Fig. 2). The choice dimension of food literacy was associated with decreased appetite but not with the two other restrictive eating behaviours, namely, picky eating behaviours and fear of negative consequences.

Stepwise regression analysis was conducted with total NIAS score as the dependent variable and age, BMI, disease phenotype, disease activity, FLEQ-Ch scores, food planning and management, selection and attitude as the independent variables. These three models (Table 4) were built by adding variables to the previous model at each step to determine whether the newly added variables would improve the proportion of explained variance of the dependent variable by the model (improvement in adjusted R^2^). In the first model, the disease phenotype was considered as predictor factor; in the second model, food planning and management scales were added, followed by disease activity in the third model. The factors relevant to the NIAS scores are listed in Table 4. As important factors in the first model, UC (β =  − 0.394) contributed negative factors to the NIAS scores. The R^2^ (adjusted R^2^) in the stepwise regression analysis was 0.156(0.153). The VIF of the variable in this stepwise regression was 1.000. In the second level of the model, food planning and management also had an impact on NIAS scores. The R^2^ (adjusted R^2^) in the stepwise regression analysis was 0.242 (0.238), and the VIF of the variable in this stepwise regression was 1.032. The subjects who had low scores of food planning and management (β =  − 0.299) may have improper eating habits. UC, food planning and management and remission were all the significant factors of the third model. This finding showed that disease phenotype (UC, β =  − 0.298), disease activity (stationary phase, β =  − 0.279) and food planning and management dimensions (β =  − 0.275) negatively predicted 31.5% of the variance in NIAS scores. The R^2^ (adjusted R^2^) in the stepwise regression analysis was 0.315 (0.310). The VIF score of 1.036 and Durbin–Watson of 1.707 indicated the lack of collinearity among the independent variables in this regression. The stepwise method was used to simultaneously remove variables that were weakly correlated with the dependent variable. These variables were removed from the final models, and the models were re-estimated for the remaining predictors. Thus, the final variables that remained in the model can comprehensively explain the distribution (Table 5).

**Table 4:** Stepwise linear regression model of predictors of the NIAS scores of patients with IBD (n = 372)

Independent variable	Model 1	VIF	Model 2	VIF	Model 3	VIF
Disease phenotype (Ref: Crohn’s disease)						
Ulcerative colitis	− 0.394 (*p* < 0.01)	1.00	− 0.342 (*p* < 0.01)	1.032	− 0.298 (*p* < 0.01)	1.058
Food planning and management			− 0.299 (*p* < 0.01)	1.032	− 0.279 (*p* < 0.01)	1.038
Disease activity (Ref: active)						
Remission					− 0.275 (*p* < 0.01)	1.036
*F*-value (*p*-value)	68.186 (*p* < 0.01)		59.044 (*p* < 0.01)		56.469 (*p* < 0.01)	
R^2^ (adjusted R^2^)	0.156 (0.153)		0.242 (0.238)		0.315 (0.310)	
Durbin-Watson					1.707

**Table 5:** Multiple regression model predicting the NIAS scores of patients with IBD (n = 372)

Variables	Unstandardized regression coefficient (*β*)	Standardized regression coefficient (*β*)	*t*	*p*
Ulcerative colitis (Ref: Crohn’s disease)	− 4.836	− 0.298	− 6.728	< 0.01
Food planning and management	− 0.439	− 0.279	− 6.348	< 0.01
Remission (Ref: active IBD)	− 4.500	− 0.275	− 6.255	< 0.01

R^2^ = 0.315, Adjusted R^2^ = 0.310; F = 56.469, *p* < 0.01; Durbin-Watson test = 1.707; Dependent variable: NIAS score

## Discussion

This study aimed to examine the prevalence and food literacy levels of ARFID in Chinese patients with IBD and to determine the associated effects of gender, age, BMI, disease phenotype, disease activity and food literacy levels on ARFID.

Our study found that 32.53% of patients with IBD from four large tertiary care centres in Nanjing, China were at risk of ARFID. The NIAS tool was selected to screen for restrictive eating behaviours study primarily because it measures the domains of eating behaviours driven by fear and appetite changes, both of which are common in IBD. The apparent overlap between the symptoms of ARFID and those of IBD, particularly the fear of eating-induced intestinal symptoms, may make it particularly difficult to diagnose in patients with ARFID. In our sample, 36 active patients had a positive ARFID score on the NIAS with a fear of negative consequences score.NIAS score is associated with CD activity periods. We found a higher proportion of participants with a positive ARFID risk when GI symptoms were active compared with that in participants without symptoms. A previous study has shown that IBD symptoms such as pain, spasticity and diarrhoea can adversely affect dietary intake, and patients avoid food more during active disease than in remission [39]. Admittedly, patients with ARFID in the active IBD will describe restricting themselves to small amounts of ‘safe’ foods, often in very small quantities [31]. Gastrointestinal disorders are an independent influence on ARFID [32]. We hypothesise that many individuals with ARFID symptoms in our sample developed ARFID symptoms by attempting to manage their gastrointestinal symptoms by avoiding/restricting food. Cross-sectional studies of the IBD population found that 49–90% of patients avoided or restricted food, and restricted diets were common in patients with inactive disease [12, 13, 33, 34]. Studies on the general population usually use a cut-off of 24 points for a positive screen NIAS questionnaire. Another pilot study performed at a different centre found a prevalence of 17% while using the 24-point cut-off [35].However, a positive score on any NIAS subscale (≥ 10 NIAS-picky eating, ≥ 9 NIAS-poor appetite, and ≥ 10 NIAS-fear of negative consequences)threshold chosen for the present work may be exaggerated the prevalence of ARFID in the IBD population. Patients with IBD may associate certain foods with their symptoms, while they often resort to dietary restriction due to the difficulty of reaching a clear consensus among specialists on a dietary plan for these patients, as well as the patients' own lack of dietary knowledge and great uncertainty about dietary recommendations [7].Therefore, clinicians should be cautious in diagnosing ARFID in patients with a clinical diagnosis of IBD. ARFID may represent a distinct subgroup of patients with symptoms of IBD who avoid/restrict food, resulting in medical consequences and/or psychosocial impairment [35].More research is certainly needed to examine the etiological and mechanistic relationships between IBD symptoms and ARFID symptoms [36].

We found that food literacy deficits were prevalent in the IBD population in China. The strong relationship between food literacy and restrictive eating behaviours is a novel and understandable finding in this study. The term ‘food literacy’ describes the concept of proficiency in food-related skills and knowledge. Food literacy refers not only to the ability needed to access and understand nutritional information, but also implies the ability to apply information about food choices and to think critically about the impact of food choices on individual health and society [37]. It is based on a more holistic understanding of healthy eating behaviours. Food literacy is therefore considered to be fundamental to supporting and maintaining healthy eating behaviours [23]. It plays a crucial role in shaping dietary patterns to reduce chronic disease and promote health [38]. Adequate levels of food literacy enable people to make appropriate and informed dietary choices in their specific environmental and social contexts [23]. However, no studies have assessed the degree of food literacy among Chinese patients with IBD. To fill this gap, we used FLEQ-Ch to measure the extent of food literacy in a cohort of Chinese patients with IBD [30]. Researchers found that participants with inadequate food literacy are likely to have a higher ARFID risk. Decreased appetite and restrictive eating behaviours due to fear of negative gastrointestinal experiences or increased gastrointestinal discomfort have been associated with reduced food literacy. Food literacy has been identified as a potential facilitator of healthy eating and emphasises the importance of understanding the consequences of knowledge use in the context of the broad food system [25]. A study found that an IBD population with high food literacy has many food choices and few daily life limitations [19]. Thus, the overall goal of developing food literacy skills in patients with IBD is to apply them to the food choice and decision-making process to promote healthy eating behaviours.

We predicted three factors for ARFID positivity in our regression model. The consistent associations of active symptoms, CD patients, and planned and managed food scores with positive ARFID risk highlight that all three indicators are important factors for ARFID risk in the IBD population. The presence of any one indicator should alert clinicians to consider ARFID screening. A significantly large proportion of patients with CD believed that diet triggers relapse (67% versus 53%, *p* = 0.007) and avoided certain foods to prevent relapses (77% versus 63%, *p* = 0.003) [33]. As observed in a past study, patients with CD experience more digestive symptoms than those with UC, which could explain why the former has higher NIAS score than the latter [12]. We also found a significant negative association between food literacy index and NIAS score, confirming that inadequate food literacy is associated with ARFID prevalence in Chinese patients with IBD. A previous study suggested that high levels of food literacy are associated with a strong ability to read, understand processes and apply information on food use and consumption by reading nutrition labels [40]. In a food environment where the availability of processed foods is high, an individual must be able to identify the foods and their nutritional contents and compare items to determine the healthy choices. Therefore, patients must have adequate nutritional knowledge to properly manage their disease to prevent the onset of ARFID. Food labelling is designed to provide consumers with reliable nutritional information to help them make informed food choices. The use of NIAS and food literacy tools in clinical practice may help identify restrictive dietary thoughts or behaviours, especially in patients who exhibit active symptoms and inflammation, allowing for timely dietary interventions and proactive counselling to minimise the avoidable incidence of ARFID in patients with IBD and improve their health outcomes. Therefore, nutrition training could lead to effective adoption strategies, such as reading nutrition labels, leading to autonomous nutritional choices and improved food literacy. This finding suggested that improving food knowledge and skills may be a valuable counselling strategy for preventing ARFID in patients with IBD.

This study has some limitations. NIAS and FLEQ-Ch scores were assessed using a self-report measure and were not corroborated with clinician assessment. Additionally, NIAS was developed and validated in healthy patients but was not validated in patients with IBD. Some of the NIAS entries relate to symptoms of dyspepsia, which overlap with symptoms in patients with gastrointestinal disorders, and their use in patients with gastrointestinal disorders may result in false positives, which need to be further evaluated in the future. As a consequence, the disordered eating behaviour of patients with IBD might have been overinflated in this study. Further evaluation of its applicability in patients with gastrointestinal diseases is needed. Lastly, because our sample was predominantly Chinese, the findings may not be applicable to other demographic groups. Despite these limitations, the data from this study provide a starting point for future research and can support the development of new measures to address eating disorders in the IBD population. This pilot study is the first to highlight the correlation between avoidant and restrictive eating behaviours and food literacy and provides the improved identification and management of ARFID through food literacy levels.

## Conclusions

This study identified the following factors affecting ARFID in patients with IBD: disease phenotype, disease activity and food literacy. Among these influencing factors, food literacy had a greater role in promoting healthy eating behaviours and a greater negative impact on ARFID eating behaviours. Participants with active symptoms and inflammatory CD are likely to be screened for ARFID risk. A bidirectional relationship may exist among food literacy, disease activity and eating behaviours, with restrictive eating behaviours perpetuating and contributing to the development of ARFID disease. These three components can be used as a reliable and convenient construct to predict the risk of ARFID and promote healthy dietary behaviours in patients with IBD.

## Data Availability

The data presented in this study are available on request from the corresponding author. The data are not publicly available due to ethical restrictions.
